# A Procedural Learning Framework for Co-creating Sponge Solutions across Scales: Supporting Integrated Management of Water-Resilient River Landscapes

**DOI:** 10.1007/s00267-026-02638-3

**Published:** 2026-11-07

**Authors:** Maike Gebker, Phoebe King, Sarah Gottwald, Joanne Vinke-de Kruijf, Laddaporn Ruangpan, Ellis Penning, Kelsey Wentling, Christian Albert

**Affiliations:** 1 Institute of Environmental Planning, Leibniz University Hannover, Hannover, Germany; 2 Department of Civil Engineering and Management, University of Twente, PO Box 217, 7500 AE Enschede, The Netherlands; 3 Deltares, Boussinesqweg 1, 2629 HV Delft, The Netherlands

**Keywords:** Climate change, Co-learning, Integrated river basin management, Nature-based solutions, Planning framework, Reflexive monitoring

## Abstract

Under the pressures of climate change, floods and droughts are increasingly challenging global water systems, requiring transformative approaches for the integrated and adaptive management of water-resilient river landscapes. Enhancing landscape “sponge functioning”, the capacity to intercept, retain, store, and release water, is increasingly promoted, yet guidance remains fragmented between local measures and landscape-scale strategies, limiting the coordinated prioritization and implementation of water-retention interventions. This paper proposes a Procedural Learning Framework to support the co-creation of sponge measures and strategies across spatial levels. It was developed through a scoping review of process-oriented implementation frameworks and action-oriented research with stakeholder knowledge in the Horizon Europe Innovation Action project SpongeWorks. The framework includes four transformation principles: shared system understanding, multi-scale transitions, co-creation, and learning and reflexivity. It operationalizes them into interconnected building blocks for strategies and measures, and introduces guiding questions to support collaborative learning. By aligning actions across spatial levels and temporal scales, the Procedural Learning Framework contributes to more coherent and adaptive planning efforts, addressing complex social-ecological-technical challenges and advancing the integrated management of water-resilient landscapes as a transferable decision-support approach for environmental management in the EU and beyond.

## Introduction

Floods and droughts are increasing challenges driven by climate change and anthropogenic activities (Pizzorni et al. 2024). With more extreme events projected (IPCC 2023), significant impacts on ecosystems are expected, including agricultural yields, water quantity and quality, soil health, and biodiversity (Furtak and Wolińska 2023). To address these challenges, researchers highlight the need for water resilience in landscapes (Rodina 2018; Falkenmark et al. 2019; Granata and Di Nunno 2026), understood here as social-ecological river basin systems that withstand, adapt and transform (Meerow et al. 2016) to climate-induced extremes, such as floods and droughts, due to their social (e.g., economic, political) and ecological (e.g., ground and surface water, soil) conditions. A crucial feature of a water-resilient landscape is its sponge function: the ability for landscapes to intercept, slow, and store water, and to release it when needed (Penning et al. 2023; Fennell et al. 2023). Recent studies highlight the potential of, but also little knowledge about, water-retention measures (such as nature-based solutions (NbS) (UNEP 2022)) to enhance sponge function, mitigating the effects of both floods and droughts on ecosystems and society (Penning et al. 2023; Granata and Di Nunno 2026). Instead of building upon traditional environmental risk management that involved siloed work and isolated technical interventions, water-resilient landscapes require transformative change (Granata and Di Nunno 2026), embracing a holistic and nature-based approach (Palomo et al. 2021).

Integrated water resource management (IWRM) is discussed as an approach that includes environmental and socioeconomic values, and cross-sectoral coordination in river basin management, emphasizing the comprehension of the river being directly connected to the surrounding environment (Hooper 2011). Although IWRM is needed to facilitate the shift towards water-resilient landscapes, current literature provides guidance framed either generically or solely at the local level for individual measures or at the landscape level for overall strategies (Fedele et al. 2019; Magnan et al. 2020; Werners et al. 2021; Robins et al. 2025). These generic and single-level approaches can overlook the complex interactions of a landscape-wide system. Poor understanding of system dependencies, cross-scale impacts, and trade-offs can result in maladaptation, which can have unintended, immediate, or future negative effects on the intervened and connected system (Magnan et al. 2016; Higuera Roa et al. 2025). Therefore, guidance on how to co-create systemic, multi-level water management along the river and connected landscapes is essential to inform integrated environmental management, cross-scale coordination, and adaptive planning for water-resilient landscapes.

To date, integrated guidance to enhance water retention across the landscape is limited. This paper proposes a Procedural Learning Framework (PLF), guiding local and regional transdisciplinary teams of environmental management practitioners and scientific researchers to collaboratively develop sponge landscape strategies and local sponge measures by emphasizing which steps to consider in the process. Sponge landscape strategies (hereafter sponge strategies) aim for landscape-level transformations towards water-resilience, including land-use, land and water management and governance changes, expanding over the next decades. Local sponge measures (hereafter sponge measures) are short- to medium-term nature-based or technical implementations and practices aligned with the sponge strategy aims. Building upon the expertise of the SpongeWorks EU project and its three European case studies (case study descriptions in the Supplementary Information), this guidance is intended to support the prioritization, coordination, and implementation of sponge interventions across spatial scales, adaptable for diverse contexts across the EU and beyond.

As a symbiosis of a procedural *and* a learning framework, this paper provides stepwise guidance to actively co-create sponge landscapes and learn rather than to observe or assess learning. Key theoretical principles for water-resilient landscapes are explored and embedded within this framework, ensuring that the guidance facilitates these landscape transformations whilst supporting the framework’s practical application of sponge measures and strategies. Guiding questions for collaborative learning are presented and function as a process monitoring and learning tool to enable conscious management decisions in advance, reflective adjustments along the co-creation, and reflexive discussions to evaluate the process after the implementation. Finally, the PLF proposes which activities are of high value for cross-level management and provides a transferable decision-support approach on how to achieve a systemic landscape transformation.

## Methodology

**Fig. 1:**
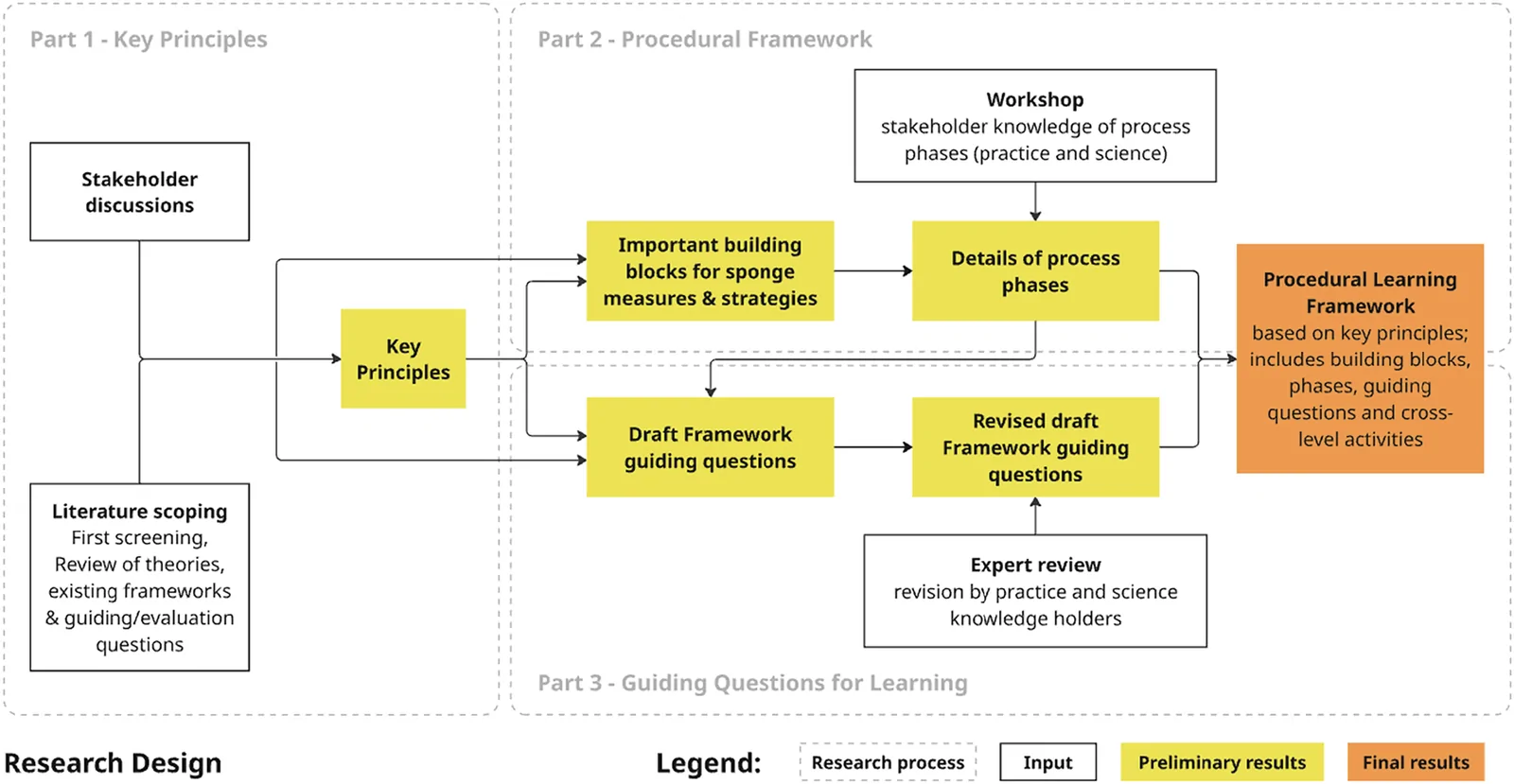
Research design is divided into developing the “Key Principles” (Part 1), the “Procedural Framework” (Part 2) and the “Guiding Questions for Learning” (Part 3). Own Figure

Figure 1 presents the methodological approach, starting with an analysis of existing frameworks for water-retention measures (Raymond et al. 2017; Beek et al. 2022; de Lima et al. 2022; Wren et al. 2022; Collier et al. 2023) and strategies (Nesshöver et al. 2017; Kumar et al. 2020; Albert et al. 2021; Beek et al. 2022; Wickenberg 2024). Those frameworks were reviewed for proposed phases and detailed procedural information. The content and order of the phases were compared and synthesized (Supplementary Information: Table SI1). This synthesis has built the foundation for a first draft of the framework. Simultaneously, to identify stakeholders’ process knowledge, this research follows an action-oriented research approach in the context of the EU project SpongeWorks^1^. The SpongeWorks consortium brings together academic and societal partners to co-create strategies and plan, implement, monitor and evaluate sponge measures implemented across three river basins. In this project, the authors act as knowledge brokers to collect data from scientific and practice knowledge sources, reflect stakeholders’ knowledge demands, translate this into scientific questions and facilitate joint learning (Vinke-de Kruijf et al. 2022). While the framework was informed by SpongeWorks’ practitioners working within case studies (Pinios in Greece, Lèze in France, Vecht across the Netherlands and Germany), the framework is aimed at being widely generalizable, thus includes no explicit case study analysis. Internal SpongeWorks meetings exposed stakeholders’ expectations, needs and barriers to establishing transformation for water-resilient landscapes. From here, key principles for landscape transformation derived from recurring themes in stakeholder discussions and the scoping review guide the practical application of the PLF.

**Fig. 2:**
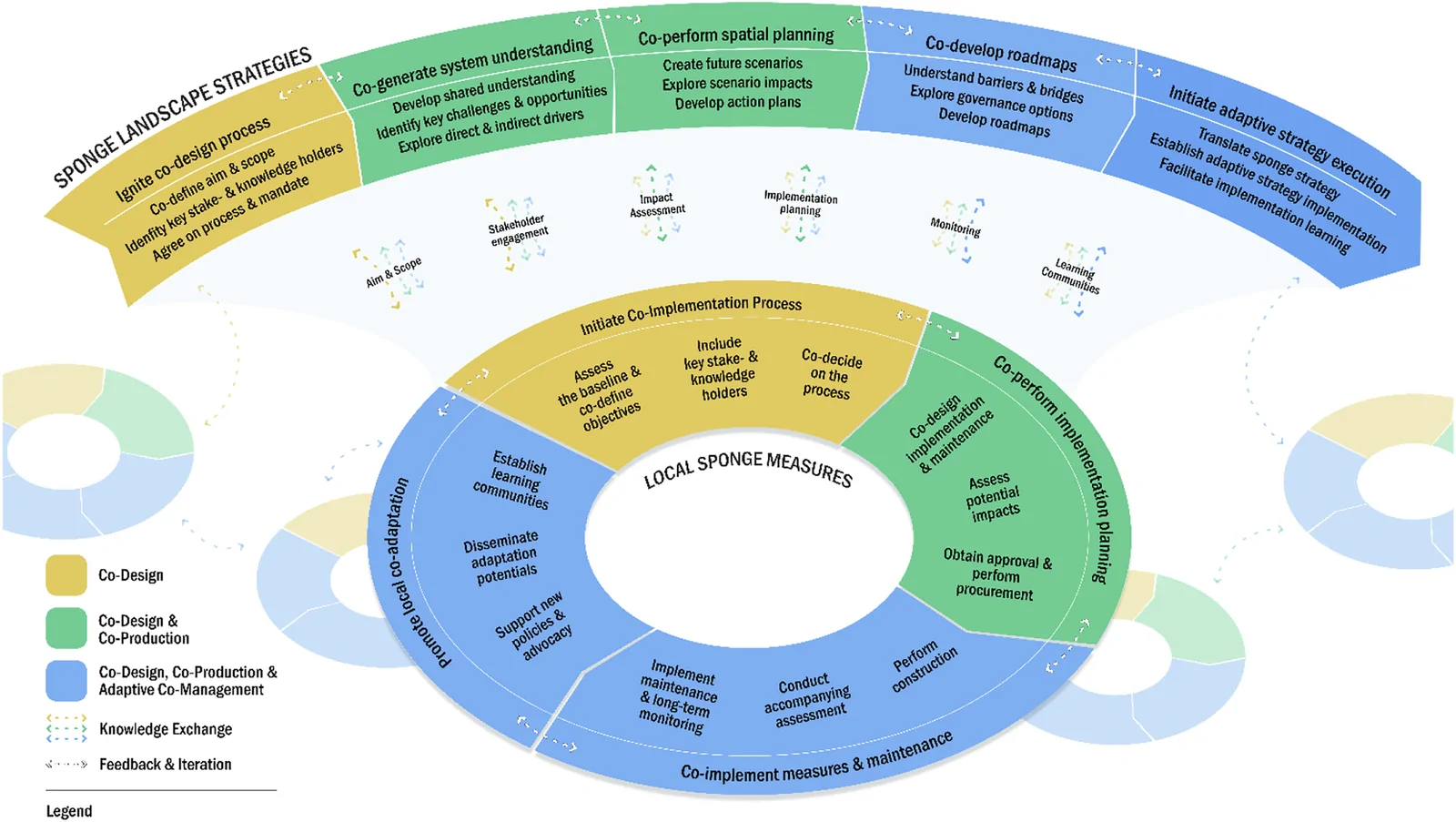
Procedural Learning Framework – Top: Sponge Landscape Strategies building blocks: Block 1 - Ignite co-design process, Block 2 - Co-generate system understanding, Block 3 - Co-perform spatial planning, Block 4 - Co-develop roadmaps, Block 5 - Initiate adaptive strategy execution. Bottom: Local Sponge Measures building blocks: Block 1 - Initiate co-implementation process, Block 2 - Co-perform implementation planning, Block 3 - Co-implement measure and maintenance, Block 4 - Promote local co-adaptation. The blocks are categorized in the overlapping approaches of co-creation: Co-design, Co-Production, and Adaptive Co-management. The yellow, green and blue arrows between sponge landscape strategies and local sponge measures show the need for high (long arrow) and moderate (short arrows) exchange. Arrows to the other local sponge measures (small circles) indicate the importance of collaboration and exchange. The white arrows show the need for feedback and iteration. Own Figure

The framework analysis was synthesized along with the key principles and refined with observations of the SpongeWorks strategy and measure planning process into building blocks and phases within a first PLF draft (final PLF shown in Fig. 2).

In part two of Fig. 1, the first draft of the procedural framework was discussed in a SpongeWorks internal online workshop to co-identify transformational knowledge (Pohl and Hirsch Hadorn 2008). Feedback from local (*n* = 14) and landscape-level partners (*n* = 14) of the three case study basins, as well as scientific perspectives (*n* = 12), was collected. The partners covered multiple stakeholder groups, including practitioners implementing sponge measures, landowners, local authorities, municipalities, and water management institutions. Those workshop participants were divided into three groups (landscape, local and scientific; 13 participants per group) to spark discussions in their field of expertise. In these sessions, the drafted framework was displayed, and the participants were asked to list the essential steps in each building block (Fig. 2) in co-creating sponge strategies and measures. Outputs from this workshop (Table SI2; SI3) complement the literature findings with practical process knowledge (e.g., to work with local ambassadors for agriculture or governance to gain trust from other practitioners) to provide a comprehensive assessment of the procedure for co-creating sponge measures and strategies. Following Hakkarainen et al. (2022), the building blocks were categorized into overlapping co-creation approaches: co-design, co-production, and adaptive co-management. The final procedural framework was analyzed according to cross-level activities enabling well-adapted, co-created sponge solutions.

In part three (Fig. 1), we developed a set of guiding questions for collaborative learning. They are inspired by the IUCN self-assessment^2^ questions and the performance questions of Sowińska-Świerkosz and García (2021) but are aligned with the procedural framework’s building blocks and phases to identify guidance, knowledge and techniques for co-generating a holistic understanding of sponge development procedures. The questions were informed by a second SpongeWorks internal online workshop. Project partners (*n* = 14) were invited via targeted recruitment according to their process expertise (e.g., coordination and stakeholder engagement, implementation, monitoring, dissemination of learnings). They were divided into four groups (each 3–4 participants) to focus on either the sponge strategy or measure development. After reading short descriptions of each phase, they indicated the most important action to learn within the phases of each building block (e.g., “ignite co-creation process” with three phases, followed by the other building blocks), first individually and then in collective discussions. Their results were later summarized by the authors, compared with the literature and phrased in reflexive guiding questions. After one additional feedback loop, the guiding questions were finalized.

Finally, the procedural framework and guiding questions were assessed for their contribution to the key principles (Tables 1 and 2) and, thus, transformative change for developing water-resilient landscapes.

### Ethical Statement

The research was conducted in accordance with the ethical standards of the EU project SpongeWorks, which includes publishing research results. Data collection was limited to consortium partners during internal workshops, involving no personal data.

## Results

### Key Principles for Contributing to Landscape Transformation

The synthesis of scientific and stakeholder knowledge revealed four key principles contributing to landscape transformations: (1) shared system thinking, (2) multi-scale transitions, (3) co-creation, and (4) learning and reflexivity.

1. Shared system thinking
  Climate change is not limited to single regions or industrial sectors, generating a set of holistic and interacting challenges. Sponge measures and strategies intervene in the social-ecological-technical system of river landscapes (SETs), affecting social, ecological and technical components across nested temporal and spatial levels (McPhearson et al. 2022). This underscores the necessity for a “common understanding of multi-functional solutions” (Nesshöver et al. 2017: 1223). Fostering common understanding of SETs towards transformative, water-resilient landscapes, it is imperative that (1) the conditions and needs that habitats and species require to fulfil sponge functioning are well understood (Wiering and Arts 2006; de Groot and de Groot 2009; Robins et al. 2025), (2) stakeholders are willing to change long-established practices (Berhanu et al. 2025), and (3) existing and planned technical solutions or infrastructures enhance the transformation towards resilient river landscapes. System transformation is primarily determined by three related attributes: the ability to withstand unexpected shocks (resilience), the capacity to influence the system’s resilience (adaptability), and the potential to develop alternative future systems (transformability) (Holling and Gunderson 2002). Transformational interventions can target systems’ leverage points on different levels, ranging from deep to shallow (shallow: e.g., changes in subsidies, feedback opportunities; deep: power to self-organize, changing underlying values of stakeholders) (Meadows 1999; Abson et al. 2017).
  Following this key principle, a PLF must acknowledge the need for increased stakeholder awareness of the systemic entanglements within their river landscape while understanding contextual drivers. By doing so, stakeholders will be able to understand the causes of local challenges and how to collaboratively overcome them by identifying interventions at different leverage points. Once developed in the beginning, the framework must enable this systems-thinking approach throughout the entire process for both sponge strategies *and* measures.
2. Multi-scale transitions
  Shared system understanding leads to the comprehension that a solution must be conceptualized on multiple spatial and temporal scales, e.g., location (landscape and local), governance (water authorities and municipality) and time (short term and expanding over generations) (Cash et al. 2006). Although higher-level governance has generally more power to change legislation, Geels and Kemp (2007) emphasize the power of niche innovations, highlighting how transitions can emerge from small-scale experimentation and adaptation (Geels and Kemp 2007). Transitions involve an iterative, adaptive approach, a set of connected changes that reinforce each other and which take place in several different areas, such as technology, economy, institutions, behavior, culture, ecology and world views/paradigms. Such transitions usually unfold over at least 25 years, reflecting the time needed for change to span roughly one generation (Rotmans et al. 2001: 167). Governing changes in those complex multi-scale systems requires dynamic steering, long-term thinking, flexible objectives, short and long-term goals, social learning and participation (Loorbach 2007) to adapt to changing conditions of external factors, such as new water management regulations.
  Leading through the multi-scale transition of water-resilient landscapes, a PLF must guide local stakeholders on the processes to implement niche innovations, and give guidance to the landscape level in governing this complex systemic change. In addition to guiding both levels, it will highlight interlinked activities between the levels to ensure streamlined planning and implementation.
3. Co-Creation
  Multi-scale transitions in complex systems require transdisciplinary and equally valued co-creation among all stakeholder groups, uniting practical and scientific knowledge (Hooper 2011; Nesshöver et al. 2017; Raymond et al. 2017; Albert et al. 2021; IUCN 2020; Beek et al. 2022). Stakeholder knowledge is of intense value in developing socio-ecological sustainable solutions in transformations (Horcea-Milcu et al. 2020) and gives scientific outcomes an even greater impact (Norström et al. 2020). However, co-creation is often challenging in terms of time and personal resources, as well as in the understanding of each other’s work and value (Norström et al. 2020). Moreover, unequal power distribution in decision-making over the co-creation process can set the path for reproducing inequality (Turnhout et al. 2020).
  Co-creation processes consist of the overlapping approaches co-designing, co-production and adaptive co-management, including co-learning (Hakkarainen et al. 2022). The latter will be described in the next section. While co-design is the starting point in which diverse stakeholders come together to conceptually plan (Hakkarainen et al. 2022), co-production is “the collaborative process of bringing a plurality of knowledge sources and types together to address a defined problem and build an integrated or systems-oriented understanding of that problem” (Armitage et al. 2011: 996). Challenges in co-production lie in addressing power relations, accepting and valuing divergent perspectives in the system, and agreeing on a shared understanding of challenges and the goal (Armitage et al. 2011). While aiming for societal and scientific impact, co-production is context-specific and utilizes innovative formats and methods. Therefore, integrating diverse stakeholders from several practice and research fields is essential for developing well-equipped solutions (Geels and Kemp 2007; Lam et al. 2021). To capture all potential impacts and value the knowledge of stakeholders, recognitional and procedural justice in stakeholder engagement is imperative (Hakkarainen et al. 2022; Loos et al. 2023).
  Co-creation and its challenging application through diverse foci, working structures and expert languages are given substantial attention when developing the PLF. To understand the status quo and future effects of the system, diverse stakeholders must be engaged throughout the entire process while trying to avoid fatigue (which may arise from too many meetings), initiating shared system understanding through co-production. In addition, we envision the guiding questions reflecting upon which and how stakeholders are engaged and how collaboration can be improved. Addressing the research gap of limited evaluation of co-production processes (Turnhout et al. 2020), the guiding questions will support assessing the co-creation process after the implementation and how it contributed to developing water-resilient landscapes.
4. Learning and Reflexivity
  In co-creation processes, the equal exchange and collaborative exploration of new knowledge is called co-learning, expanding across the aforementioned co-creation approaches (Hakkarainen et al. 2022; Pahl-Wostl et al. 2007). Co-learning enables stakeholders to reflexively influence their environment and change conditions towards transformative change (van Mierlo and Beers 2020). This reflexive learning supports adaptive co-management by adapting to unforeseen changes, and consequently building resilient systems (Werners et al. 2021; Hakkarainen et al. 2022; Haasnoot et al. 2024; Zorita et al. 2025). To foster co-learning and adaptive co-management of transitions across multiple stakeholder groups, engagement and interaction among stakeholders in all activities are required (van Mierlo and Beers 2020; Hakkarainen et al. 2022; Sarkki et al. 2024; Bradshaw et al. 2026). This can be achieved by reflexively exchanging on stakeholders’ system knowledge (about the problem), target knowledge (the need for change) and transformation knowledge (the actions to implement) (Pohl and Hirsch Hadorn 2008; Werners et al. 2021).
  To promote learning and reflexivity, the framework must foster multiple stakeholder collaboration, exchange on all scales, levels and activities during the process, as well as iteration and feedback adaptation. It shall embody the need for exchange and learning from others, while valuing practical and scientific knowledge equally. Guiding questions for collaborative learning should enable reflexive discussions in planning and evaluation, encouraging to find consent over conflicting views and aims.

Following these four key principles, the PLF will guide practitioners and scientists through the practical approach of co-creating sponge strategies and measures, while being aware of and considering the systemic complexity of a landscape.

### Procedural Learning Framework

The PLF (Fig. 2) demonstrates the process of co-creating sponge measures and strategies and their interactions. Each level (landscape strategies and local measures) is divided into building blocks with three phases within each block. The framework outlines key considerations in the co-creation, not prescribing a chronological sequence. Some tasks extend over a longer period (e.g., stakeholder engagement or maintenance) or the entire duration (e.g., monitoring). The application depends on spatial and governance contexts, as well as capacity and data resources. Moreover, it is an iterative process relying on exchange and feedback from other phases and the other level. For example, the aim and scope (Block 1, phase 1) will be co-defined in the beginning, but any challenges emerging while “identifying key challenges and opportunities” (Block 2, phase 2) may lead to an adaptation of the initial scope. An example of how the process could be applied sequentially is shown in the Supplementary Material (Figs. SI1 and SI2). To underscore stakeholders’ involvement across the process, the framework follows the co-creation approach (Hakkarainen et al. 2022) and is categorized in co-designing, co-production of knowledge, and adaptive co-management, including co-learning.

The guiding questions for collaborative learning foster reflexive learning by challenging common practices during the co-creation process and highlight consideration for the planning stage, establishing new learning targets (van Mierlo et al. 2010; Collier et al. 2023). As the PLF closely reflects the key principles, guiding question tables for sponge strategies (Table 1) and measures (Table 2) in each section illustrate how the phases operationalize these principles and contribute to landscape transformation towards water-resilience.

### Procedure for Developing Sponge Strategies on a Landscape Level

**Block 1: Ignite the co-design process** focuses on defining the *aim and scope* of the landscape strategy, *identifying stakeholders* involved and collaboratively *agreeing on the process and mandate*. Geographical extent, time scales and impacts are defined to agree upon objectives (Holling and Gunderson 2002; Collier et al. 2023). Embracing SET-system thinking, the strategy is coordinated with other activities in the region and all governance levels involved (Cash et al. 2006; de Lima et al. 2022). Stakeholders affected by or affecting the system are iteratively and continuously categorized and engaged to ensure equal representation (recognitional justice) and inclusive participation (procedural justice) (Loos et al. 2023). To understand stakeholder dynamics and to navigate within the system, it is important to identify ambassadors and potential opponents and address them through target-oriented stakeholder discussions and with a lot of patience (Loos et al. 2023; Reed et al. 2008). This ensures an equitable representation of their needs in the strategy. Barriers to equal participation are iteratively identified and, where possible, collaboratively addressed either through mechanisms designed to overcome them or by organizing participants into smaller groups to reduce hierarchical dynamics. Expectations and responsibilities are co-defined early on among all stakeholders (Collier et al. 2023; Bradshaw et al. 2026). The guiding questions (Table 1) are discussed to pre-consider all relevant steps and possible challenges in the process. Aiming to identify context-specific enablers and barriers, divergent perspectives are expected and valued, and underlying expectations are discussed until consent is reached. Flexible processes are established, communicated, and iteratively adapted to changing conditions (Loorbach 2007; Raymond et al. 2017; Nesshöver et al. 2017; Bradshaw et al. 2026). Financial models are established for long-term maintenance, as maladapted funding can limit aim and participation (Albert et al. 2021; Collier et al. 2023).

**Table 1:**
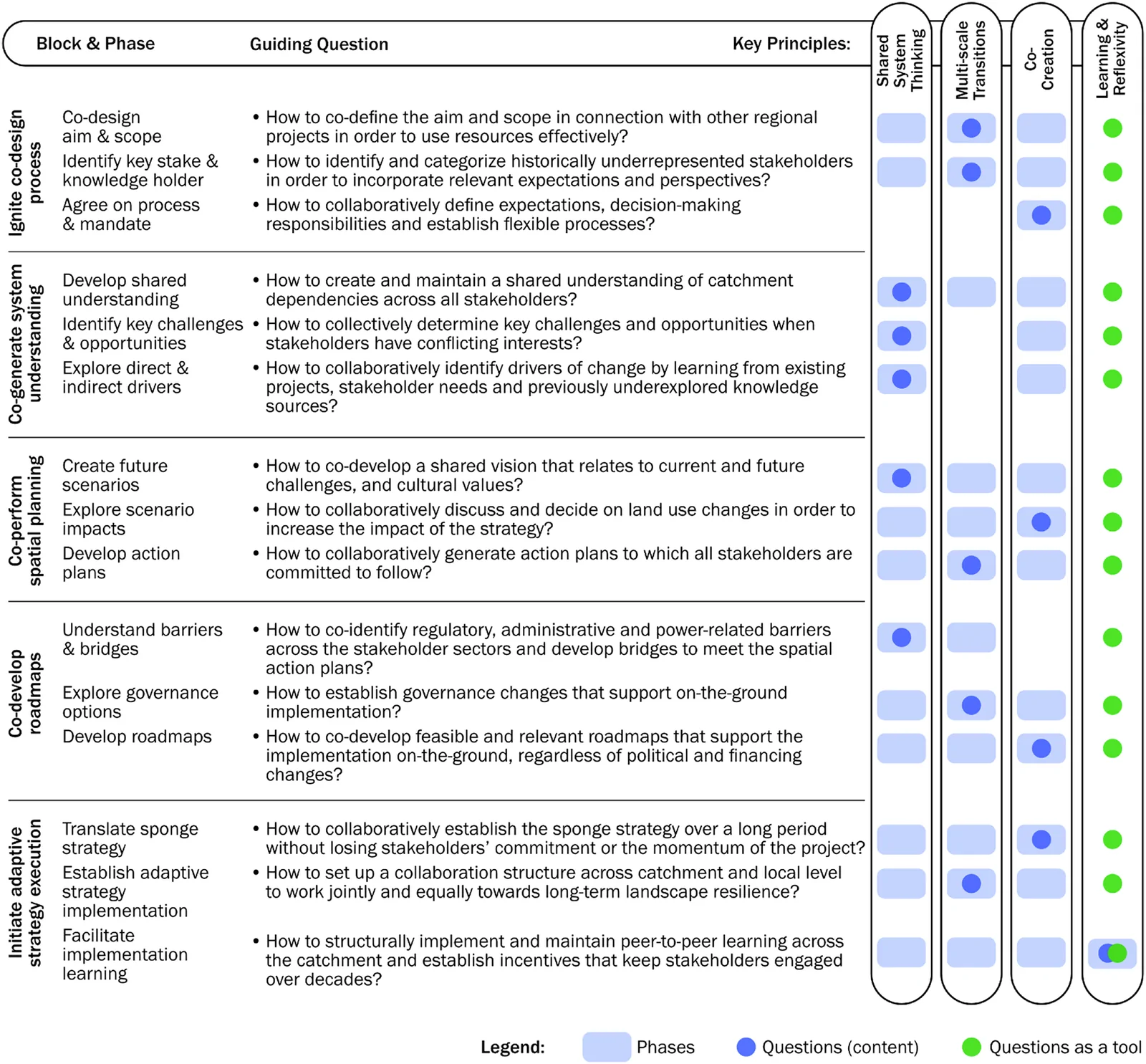
Uptake of the key principles: shared system thinking, multi-scale transitions, co-creation, learning and reflexivity in sponge strategy phases (light blue square) and guiding questions (dark blue dot). The guiding questions always aim for reflexivity and learning in their format (green dot)

**Block 2: Co-generate system understanding** requires a *shared comprehension* of the SETs dimensions, *key challenges and opportunities* among all stakeholders. Local knowledge sources are essential for identifying the status quo and entanglements among challenges and opportunities to enhance sponge functioning and for exploring potential impacts (Holling and Gunderson 2002; Raymond et al. 2017; Albert et al. 2021). Based on this comprehension, shallow and deep leverage points are assessed to intervene in the system (Meadows 1999; Abson et al. 2017). Adjustable, preliminary conclusions on potential pathways are drawn regardless of minor uncertainties to define a vision for discussion and revision (Nesshöver et al. 2017). *Direct and indirect drivers* for stakeholder motivation to implement sponge strategies are explored. Therefore, context-specific factors are assessed, such as the impacts of extreme floods in the region, as well as legal and financial incentives. Key region-specific benefits of implementing sponge strategies are identified and assessed to resonate with stakeholders’ wishes and fears (Abson et al. 2017; Nesshöver et al. 2017). To use resources effectively, stakeholders with a firm conviction of the vision function as early adapters or pioneers to develop showcase material (e.g., easy-to-implement measures) in order to try to convince those who tend to be more critical (Thaler et al. 2023).

**Block 3: Co-perform spatial planning** aims to create the future land-use and water management of the regional landscape by using visioning and action plans. Historic development, regional climate change scenarios and demographic models inform stakeholders’ discussion of landscape visions for the next decades, addressing the multi-scale transition from land-use changes to policy scenarios (Cash et al. 2006; Albert et al. 2021; Wickenberg 2024). *Co-created scenarios* are illustrated through spatial maps. *Impacts*, trade-offs, and (co-)benefits of the future scenarios on the SETs are assessed (Zorita et al. 2025). Affected stakeholders are identified to discuss compromises (Albert et al. 2021; Beek et al. 2022). These discussions result in regional, spatially explicit *action plans* that collaborate with national water management plans, showcasing how to implement the strategy in terms of land-use changes and sponge function (Beek et al. 2022; Penning et al. 2023; Haubrock et al. 2025).

**Block 4: Co-develop roadmaps** aims to explore governance and financing options and understand regulatory barriers to develop roadmaps towards water-resilient landscapes. Regulations, administrations, power relations and coalitions are analyzed with all stakeholders to *understand barriers and bridges* for the strategy and identify leverage points (Abson et al. 2017; Collier et al. 2023). *Governance options* are explored to co-learn from previous failures (e.g., siloed departments, lack of follow-through, complex administrative processes) and to better co-facilitate novel or newly combined governance approaches (van der Jagt et al. 2023). *Roadmaps* are the conceptual goal of the strategy, illustrating the adaptive transition pathways from the status quo to the implementation of the agreed action plans (Nesshöver et al. 2017; Albert et al. 2021; Haasnoot et al. 2024). They include an adaptive management approach (Beek et al. 2022) to address challenges and prevent negative impacts, and define collaborations and responsibilities among stakeholders. Effective levels for policy implementation are determined, and governance approaches with policy actors co-developed (Raymond et al. 2017; Collier et al. 2023).

**Block 5: Initiate adaptive strategy execution** starts the long-term action plan and roadmap implementation of the sponge strategy. This *translation* is a very tedious process, finding solutions for difficult land ownership, such as land consolidations. Multi-level governance changes are again discussed to align with practical implications in governance and financing (Lam et al. 2021). *Establishing adaptive cross-level strategy implementation* requires multi-level coordination with local implementation. A long-term process-monitoring plan assesses the performance and SETs impacts of governance changes and measures implementation (de Lima et al. 2022; Beek et al. 2022). To *facilitate implementation learning*, cross-landscape and cross-level learning communities are established or existing communities extended. Sharing of local knowledge, success stories and scientific evidence aims for deeper and intrinsic ownership of stakeholders’ sponge thinking (Moore et al. 2015; Collier et al. 2023; Wickenberg 2024), establishing a diverse knowledge pool for sponge strategies and increasing the replication in other regions (“out-scaling” (Moore et al. 2015)). The communication process starts early in the project to enable cross-project collaborations.

### Procedure for Developing Sponge Measures on a Local Level

**Block 1: Initiate the co-implementation process** of sponge measures, which can be triggered by events such as the storm “Daniel” in 2023 in Greece (Mavroulis et al. 2024). After these events, entities with the right of land management, e.g., farmers, landowners, businesses, NGOs and local or regional authorities, collaborate with key stakeholders, such as neighboring farmers, to identify the problem (Wren et al. 2022). A *baseline assessment* is essential to set the objectives of the measure. In case no prior monitoring data exists, indicators must be defined according to the measure and conditions, equipment set in place, data handling, maintenance, and storage organized. This requires understandable indicators that are relatable to stakeholders to encourage their involvement, creating a foundation for future decision-making and trust and thereby enhancing co-creation (Pahl-Wostl et al. 2007). For the multi-dimensional assessment (Albert et al. 2021), a set of core and optional indicators for water, soil, biodiversity and socio-economics enables inexperienced implementers. It also acts as a readiness assessment, exposing data gaps and feasibility constraints. In parallel and in intensive exchange with the baseline assessment, measure *objectives are defined* that address site-specific water-retention challenges while acknowledging potential long-term effects. Learnings from other local water-retention activities are collected (de Lima et al. 2022). Financial resources are allocated, a partnership developed or applied for funding from third parties, e.g., loans, funds, governmental subsidies (Beek et al. 2022; Wren et al. 2022). For *including key stakeholders and knowledge holders*, proactive, multilingual strategies are established to encourage collaborative, representative, equal and just site planning (e.g., including smallholders and temporary workers). Existing local networks are exploited to build upon trust relations (Raymond et al. 2017). To *co-decide on the process*, expectations and responsibilities (Collier et al. 2023) are co-defined to enable flexibility in responding to arising challenges (Raymond et al. 2017) while everyone stays informed about decisions made. Process monitoring, including feedback adaptation through iteration mechanisms, is set in place through regular meetings and reflexive discussions, e.g., by using the guiding questions (Table 2). This is essential to identify dissent among stakeholders, the need for adjustments, and it enables learning how to improve the process, to compare expectations and performance (van Mierlo et al. 2010). Conclusions inform the adjustment of the current plan. However, the monitoring process should not request too much of the stakeholders and keep the implementation in focus (Norström et al. 2020).

**Table 2:**
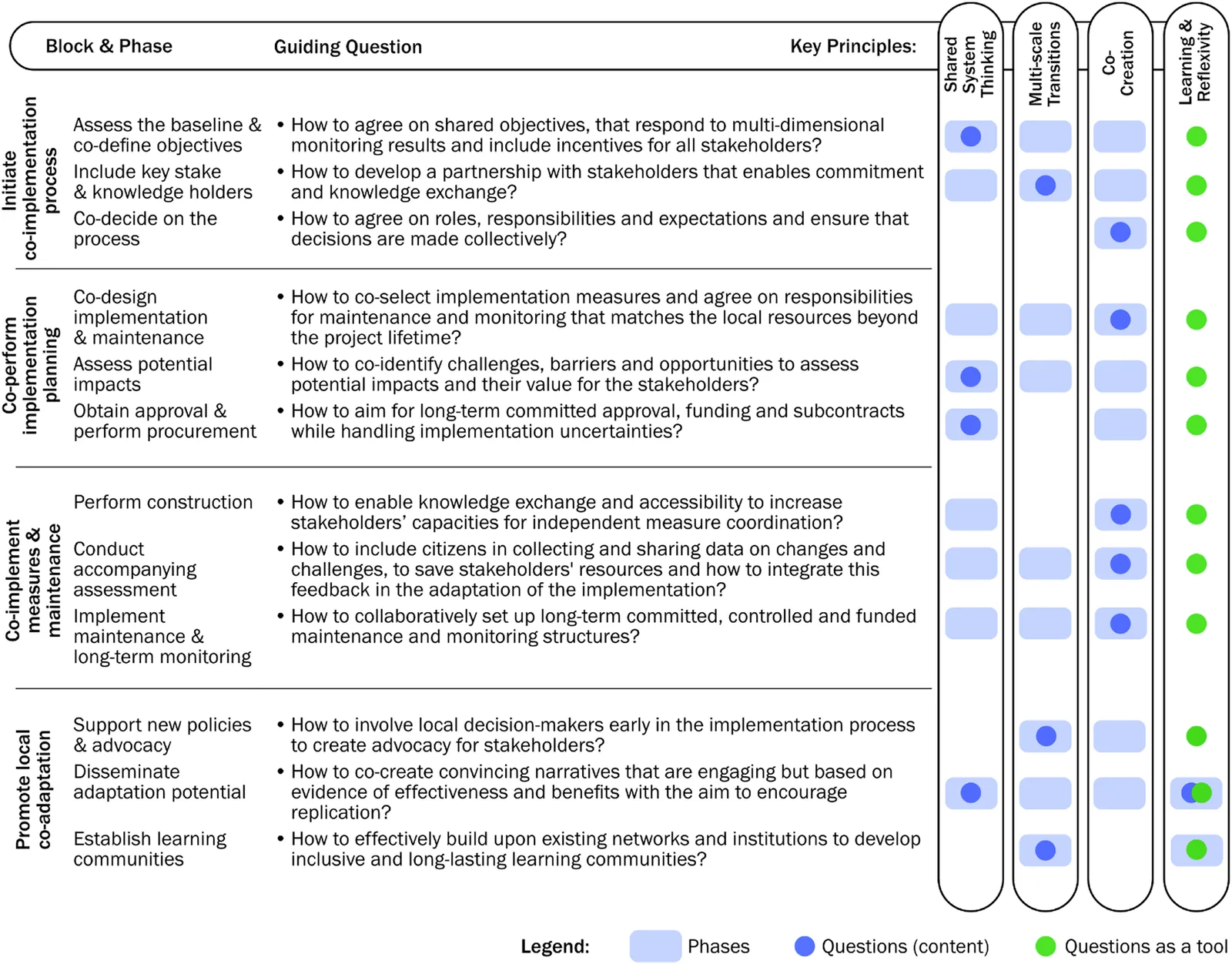
Uptake of the key principles shared system thinking, multi-scale transitions, co-creation, learning and reflexivity in sponge measure phases (light blue square) and guiding questions (dark blue dot). The guiding questions always aim for reflexivity and learning in their format (green dot)

**Block 2: Co-perform implementation planning** starts with selecting (Raymond et al. 2017; Wren et al. 2022) and *co-designing the implementation and maintenance* structures, according to local challenges. Current practices and construction plans are exploited to enable cumulative co-benefits of implementations, such as integrating measures in infrastructure construction. Maintenance can be managed by building upon existing business or volunteering models or establishing new ones (Collier et al. 2023), commencing immediately after the implementation, e.g., stabilizing sides of an infiltration basin with a grass turf and managing other vegetation (Woods-Ballard et al. 2007; Wren et al. 2022). An *assessment of the potential impacts* of the measure on the SETs (upstream-downstream interactions) is imperative. Additionally, the impact for stakeholders, such as economic and socio-ecological costs, benefits (Raymond et al. 2017) and timelines are defined, as well as barriers and opportunities to avoid unexpected challenges (de Lima et al. 2022). Non-human stakeholders (aquatic and terrestrial organisms) are equally considered. *Approval and procurement* processes are planned well in advance and depend heavily on the national/regional regulations. Plans and adjustments are communicated early and transparently to reduce institutional and governmental challenges and to enable long-term agreements between stakeholders and the institution of approval or procurement (Wren et al. 2022).

**Block 3: Co-implement sponge measures and maintenance** focuses on *performing the construction*, which depends heavily on the chosen solution (Woods-Ballard et al. 2007; Wren et al. 2022). In some cases, subcontractors are hired to perform the construction. If no actionable knowledge is present, private implementers require greater support from the network to avoid negative experiences and therefore abortion of the implementation (Raymond et al. 2017). In parallel, a*ccompanying assessments* during implementation are used to identify changes and immediate challenges to adjust according to the objective (Collier et al. 2023). The comprehension of slow-rising effects of the implemented measures is imperative to maintain motivation and commitment by the stakeholders. It follows the *implementation of maintenance and long-term monitoring* structures, deriving from those previously planned and entails precise maintenance schedules with management and operational responsibilities (Collier et al. 2023). Maintenance is closely interlinked with monitoring performance and adjusting accordingly. Nature-based measures require a long settling time before full function is achieved, with stable performance after 10 years (Lehmann et al. 2025). Long-term monitoring is established as a regular mechanism, at best, with little effort for the executing stakeholders.

**Block 4: Promote local co-adaptation** targets the greater landscape transformation and increases the uptake of sponge measures. Local decision-makers are invited to the implementation planning early on, to experience implemented sponge measures, and learn from measure effectiveness to generate bottom-up transitions (Geels and Kemp 2007). Local implementers must rely on their networks and well-connected partners to reach local decision-makers. Narrative storytelling of the effectiveness evidence supports the *dissemination of adaptation potential* to other regions. Sharing these and other learning materials (e.g., handbooks) on stakeholder-relevant multimedia channels (e.g., videos, social and local media) can increase the replication and adaptation to different contexts (out-scaling potential (Moore et al. 2015)). Ambassadors, such as trusted stakeholders, farming advisors and other well-connected intermediaries, can increase regional out-scaling intensively (Collier et al. 2023). Existing networks are exploited early on to *establish learning communities*. Learning events, field visits and mentorships enable practitioners to learn from implemented measures (Collier et al. 2023), supporting not only learning but also the change of cultural norms (e.g., “we have always done it this way”) (Moore et al. 2015).

### Linkages between Planning Levels

Due to differences between local and landscape levels, the spatial planning for multiple societal goals and water management within the landscape is a complex activity, affecting the entire system. By making the soil and water system a fundamental underlying governing planning layer, this framework guides the discussions on increasing sponge functioning on both levels. The long-term sponge strategy aims to improve resilience towards water-related landscape challenges. However, it is not limited to governmental activities, as it includes multiple local measures at different sites and times to target the transformation step-by-step (Geels and Kemp 2007). Moreover, the local measures are expected to have a greater impact when planned as part of the overarching strategy towards water-resilient landscapes to address floods and droughts, taking upstream and downstream interactions into account (Higuera Roa et al. 2025). Revisions and adjustments at both levels are required (Haubrock et al. 2025) to steadily reduce the gap between current outcomes and the target of water-resilient landscapes. Here, we highlight the interlinked activities between the sponge strategy and measure development in the co-creation approaches.

#### Co-Design

While co-designing sponge strategies and measures, the *aim, scope* and *stakeholder engagement* link the activities across both levels. The *aim* of the measures is in line with those of the strategy to address the sponge function as well as current and future land use and management (Beek et al. 2022). To achieve water-resilient landscapes, all activities follow a specific roadmap. Spatial maps facilitate discussions on aligning the strategy with societal and economic impacts. The strategy’s roadmap accounts for diverse landownership realities and their implementation of local measures. Thus, private landowners and managers, as well as the affected public, are key *stakeholders to engage*. Stakeholders, including local farmers, landowners, other implementing perspectives, or chosen representatives (Bradshaw et al. 2026), are invited to the strategy discussions, ensuring the integration of practical implications of governance decisions and co-decision-making. Although those stakeholders will have different foci, consent needs to be found. Similarly, governmental stakeholders are actively informed about local activities early on to facilitate bureaucratic processes in approval requests and to disseminate local experiences.

#### Co-Production

Within co-production, *impact assessment* and *implementation* link the two levels. New sponge measures have an impact on the entire landscape, affecting the ecosystem in all dimensions (groundwater-, soil-, terrestrial- and aquatic ecosystems, upstream and downstream interactions and societal dimensions). Equally, decisions made in the strategy planning may have an impact on the daily lives of local stakeholders as well as downstream regions’ water management, ecosystems and industries. Potential impacts are considered at the beginning of the planning phase to limit negative consequences. For effective *implementation planning*, upcoming construction and land use/management changes are reported to the governance bodies developing the strategies. In that way, water-retention solutions can be implemented at planned construction sites (e.g., on roads, parks) and therefore increase the number of sponge measures. Feedback for adjusting the strategy and measures is considered at both levels.

#### Adaptive Co-Management

In adaptive co-management, *monitoring* and the *establishment of learning communities* connect the local and landscape activities. Monitoring is not limited to hydrological, soil, biodiversity or socio-economic indicators changed by the implementation of a measure. It includes monitoring of the process, collaboration and learning. In adaptive co-management of sponge landscapes, expectations, capacity and feasibility are monitored and coordinated with the local level. Unexpected impacts of measures are discussed at the landscape level to adjust implementation actions. The adaptability to project changes affects the resilience of the solution, e.g., in terms of long-term governance. Ecological, societal, technical and economic changes on one level will have immediate to long-term impacts on the other. To coordinate the exchange of new findings or reflections, *learning communities* (Zamiri and Esmaeili 2024) are established, including local and landscape stakeholders. These support the institutionalization of stakeholder participation, which enhances successful engagement processes (Reed et al. 2008). They bring together stakeholders from several measures within the same landscape and between different landscapes. This enables collaborative learning about measures, implementation challenges, maintenance structures, opportunities, and overcoming barriers, offering experience exchange and boosting stakeholders’ confidence in the process. Furthermore, it supports learning about the greater picture of water-resilient landscapes and how land-use changes can contribute to it.

Given the temporal difference in the development of sponge strategies and measures, it is imperative to emphasize that the implementation of sponge measures at a subsequent stage of the strategy is linked to the ongoing process and aligns with collective learning outcomes. Consequently, the objectives and scope of the sponge measures are synchronized with the adapted aims and scope of the strategy.

## Discussion

### The Procedural Learning Framework Supports Transformations Towards Water-Resilient Landscapes

The key principles for landscape transformation highlight the conditions that must be established to facilitate well-adapted sponge solutions. In the PLF, they are operationalized via coordinating implementation planning across multiple scales (e.g., governance, location, duration), inclusively engaging all affected entities across the entire process, facilitating co-creation from expectation management to maintenance, and enabling reflexivity through iterative discussions. In doing so, they counteract drivers of maladaptation, such as unconsidered trade-offs and inadequate resources in administration (Higuera Roa et al. 2025), while contributing to transformative adaptation in the complex system of SET landscapes (Magnan et al. 2020; Werners et al. 2021; Biesbroek et al. 2026).

The PLF demonstrates how to manage well-adapted sponge strategies and measures by co-creating water-resilient landscapes. The linkages between levels emphasize activities highly relevant to cross-level collaboration and environmental management decision-making (aim and scope, stakeholder engagement, impact assessment, implementation planning, monitoring and learning communities). The cross-level collaboration enables the co-production of system understanding by the inclusion of diverse stakeholder perspectives, as well as the assessment and monitoring of landscape-wide impacts. Local measures planned in isolation address drivers of maladaptation only partly, which can lead to unintended, negative effects on society and ecosystems, and exacerbate the risks of climate change (Magnan et al. 2016; Higuera Roa et al. 2025). Co-created strategies for adaptation, as presented in the PLF, address long-term impact and climate risk reduction by conceptualizing measures according to one overarching strategy. The building blocks and phases ease the long-term process by facilitating small-step strategy development and decision-making and the coordinated prioritization of interventions across scales (Magnan et al. 2020; Haasnoot et al. 2024). This approach promotes collaborative learning, building capacity for adaptive planning and for desirable futures, which contributes to transformative adaptation planning (Werners et al. 2021). Providing this innovative and path-shifting guidance, the PLF facilitates restructuring local and regional planning towards transformative landscape adaptation (Fedele et al. 2019).

The PLF can support other planning frameworks and practices in guiding the development of multi-scale, integrated policy solutions and regulations. Room for the Rhine^3^, the Emscher restoration^4^, the Danube restoration in the south of Vienna^5^, the Denmark tripartite arrangement, and the Kvorning area^6^ have already implemented long-term transformative adaptation projects that are strongly supported by governmental policies and regulations. In the Netherlands, a National Climate Adaptation Strategy (MINIENM 2016) has been established to coordinate among regional and local solutions. In enabling multi-stakeholder co-creation and acknowledging the value of non-scientific knowledge, the PLF prevents siloed policy development, supporting solution options for sustainability challenges (Lang et al. 2012). It brings together local implementers and regional decision-makers at an early stage, building up relations and facilitating reflexive co-learning, enabling groundwork and policy alignment. The PLF promotes step-by-step strategy planning, including parallel implementation of measures that can support governmental decision-making and uptake of adaptation development, as first quick-wins and co-benefits can be identified within the process (Magnan et al. 2020; Haasnoot et al. 2024). To avoid new regional adaptation plans after each change of government, sponge strategies outlined in the PLF are planned to be adjustable and respond to current conditions while not losing track of the overall aim of landscape transformation. International regulations, such as the Water Framework Directive, aim for adaptation strategies on multiple spatial and governance scales (EC 2000). However, international policies lack stepwise guidance to reduce the barrier for small-scale implementers and authorities. The PLF contributes to the objectives of international policy regulations by supporting the uptake and, therefore, context-specific repetition of sponge measures and strategies to co-create water-resilient landscapes under varying governance contexts.

The PLF may contribute to water-resilient landscapes by enabling the out-scaling of sponge strategies and measures to multiple regions. Establishing learning communities and fostering exchange with other regions at both levels disseminates the newly learned knowledge and enables others to replicate (Moore et al. 2015; Sarkki et al. 2024). The guiding questions steer reflexive discussions within a group to initiate the sponge strategy and measure co-creation. The PLF mainstreams sponge solutions and lowers the barrier for initiation by providing this stepwise approach and fostering adaptive governance (Raymond et al. 2017; King et al. 2023). So far, adaptation planning has been mostly developed for one region, ignoring the fact that rivers cross borders, making water retention a transboundary objective (Timmerman et al. 2017). The PLF is informed by a cross-border strategy development in SpongeWorks, therefore providing suitable guidance for other transboundary strategies to adopt. However, following varying national regulations and governance structures creates further challenges.

### Governance Challenges Affecting Procedural Learning Framework Uptake

The implementation of the PLF in practice faces several obstacles. First, the uptake of new values and long-term future thinking takes time, which is especially difficult for collaboration between multiple stakeholder groups (Haasnoot et al. 2024). Convincing municipal decision-makers can be especially challenging because strategy development takes longer than governmental mandates. The contradiction of limited capacity and the time it takes to open up new learning practices is challenging for establishing the guiding questions as a reflexive planning tool. Furthermore, the realization of inclusive and just co-creation adaptation projects requires time from all involved parties. Running alongside daily business, especially for practitioners and government stakeholders, time and capacity asymmetries can arise among stakeholders (e.g., implementers vs. authorities vs. researchers), which can constrain co-production and continuity of engagement. In SpongeWorks, this constraint was experienced in co-designing and establishing appropriate, long-term monitoring networks. The PLF counteracts this obstacle by advising on initiation meetings with small-group discussions on expectations, responsibilities and term definitions, co-generating understanding of existing perspectives in the system. Although such meetings take a lot of time in preparation, this baseline comprehension encourages collaboration and makes it easier to collectively overcome emerging challenges.

Second, power asymmetries among stakeholders can limit the inclusivity of co-creation processes (Wiering and Arts 2006; Berhanu et al. 2025). Dominant stakeholders and decision-makers can block the bottom-up and peer-developed adaptation processes (Fedele et al. 2019). The exclusion of less powerful stakeholders risks overlooking relevant challenges and unintended consequences (Loos et al. 2023; Higuera Roa et al. 2025). To overcome this challenge, the PLF includes continuous stakeholder categorization, aiming for balanced engagement of diverse power levels. Opponents are identified at the beginning and engaged with to address their values, needs, expectations and priorities. If conflicting discussions arise, the involved stakeholders and the neutral coordination role should take time to identify underlying reasons, e.g., trade-offs and costs. In cases of uneven power relations, professional facilitators can support inclusive and equal participation (Reed et al. 2008).

Third, the PLF promotes experimental and adjustable objectives, responding to changing conditions. This contradicts many funding and approval applications, requesting detailed management plans on implementation and impact. Options to deviate from proposals are often limited. Also, bearing costs after project lifetime is a great challenge in implementation, e.g., for implemented monitoring networks, online platforms, and overall maintenance. To overcome these obstacles, the PLF advises developing alternative funding options, such as building upon other business models, developing new ones or establishing funding partnerships. A bottom-up example is the Environmental Farmers Group in the UK, supporting farmers with natural capital trading and fair financing rewards^7^. Additionally, existing projects are working on platforms to share suitable funding options for measure implementation (e.g., Pathways2Resilience^8^). These constraints highlight the importance of embedding adaptive management flexibility within implementation and funding arrangements.

### Future Research

While this research was informed by the SpongeWorks project experiences, the framework is primarily based on literature to provide general guidance. Stakeholder inputs derived from activities in the case studies are therefore context-specific. However, applying the framework through a case study analysis would increase the level of detail of practical implications. Implementing the framework in different biogeographical regions and comparing it with regions that do not apply the framework may draw out variations in effectiveness and could reveal new learnings. To foster out-scaling of sponge strategies and measures, an identification of success and failure factors, enablers and tools is needed. Finally, the performance should be further elaborated across other socio-political, ecological settings, capacity levels and contexts to assess its robustness as a transferable environmental management approach.

## Conclusion

The PLF contributes to integrated environmental management and water-resilient landscapes by providing key principles, procedural guidance, and reflexive learning questions to support the coordinated development of landscape sponge strategies and local sponge measures. It emphasizes the importance of stakeholder engagement, local knowledge, and transdisciplinary co-creation, while showing how these can be integrated into the development of sponge solutions across spatial scales. Alongside the guiding questions for collaborative learning that foster reflexive discussions during planning and evaluation, the framework identifies critical linkages between activities across spatial scales. By connecting local sponge measures with long-term landscape strategies, the PLF supports more coherent and integrated approaches to water-resilient landscape transformation. As such, the PLF offers a widely applicable framework for improving the transdisciplinary planning, coordination, and implementation of sponge interventions in environmental management practice.

## Supplementary information

Supplementary Information

## Data Availability

All generated data is included in the Supplementary Information.

## Footnotes

1 SpongeWorks: https://www.spongeworks.eu/

2 IUCN Self-assessment tool: https://nbs-sat.iucn.org/

3 MERLIN: https://project-merlin.eu/cs-portal/case-study-04.html

4 Climate ADAPT: https://climate-adapt.eea.europa.eu/de/mission/solutions/mission-stories/the-emscher-restoration-story19

5 DANUBE4all: https://www.danube4allproject.eu/upper-danube

6 MERLIN: https://project-merlin.eu/cs-portal/case-study-01.html

7 Environmental Farmers Group: https://www.environmentalfarmersgroup.co.uk/

8 Pathways2Resilience: https://www.pathways2resilience.eu/finance-catalogues
