# Supplementary material for: A Procedural Learning Framework for Co-creating Sponge Solutions across Scales: Supporting Integrated Management of Water-Resilient River Landscapes
Source: Environ Manage. 2026 Nov 7;76(11):346. doi: 10.1007/s00267-026-02638-3 (PMC13744546; doi:10.1007/s00267-026-02638-3)
Supplement: Supplementary file 1 [file 267_2026_2638_MOESM1_ESM.pdf]

Supplementary Information

This Supplementary Information has not been peer-reviewed.

Title: A Procedural Learning Framework for Co-creating Sponge Solutions across Scales: Supporting Integrated Management of Water-resilient River Landscapes

Journal: Environmental Management

Maike Gebker [a*] - 0000-0003-2485-9146
Phoebe King [a] - 0000-0003-0660-3649
Sarah Gottwald [a] - 0000-0001-6784-4249
Joanne Vinke-de Kruijf [b] - 0000-0003-2691-4348
Laddaporn Ruangpan [c] - 0000-0002-7803-0600
Ellis Penning [c] - 0000-0002-9980-6930
Kelsey Wentling [b] - 0000-0001-7157-8561
Christian Albert [a] - 0000-0002-2591-4779

[a] Leibniz University Hannover, Institute of Environmental Planning, Herrenhäuser Str. 2, 30419 Hannover, Germany
[b] Department of Civil Engineering and Management, University of Twente, PO Box 217, 7500 AE, Enschede, The Netherlands
[c] Deltares, Boussinesqweg 1, 2629 HV, Delft, The Netherlands

* Correspondence to: gebker@umwelt.uni-hannover.de, +49 511 762 2697 (Maike Gebker)

List of content

SpongeWorks Case study description

Tables

Table SI1 Literature scoping for procedural steps in developing catchment sponge strategies

Table SI2 Literature scoping for procedural steps in developing local sponge measures

Table SI3 Workshop 1: Sponge Landscape Strategy results of key activities in each phase

Table SI4 Workshop 1: Local Sponge Measures results of key activities in each phase

Figures

Fig. SI1 Example of a sequential development process for sponge landscape strategies

Fig. SI2 Example of a sequential development process for local sponge measures

SpongeWorks Case study description

SpongeWorks is an EU Horizon Research and Innovation action, analyzing the implementation of sponge landscapes at the local and landscape level in three different case studies. They differ in geographical, social and bureaucratic contexts, spreading across four different countries, facing different climatic and hydrological challenges. The following information is a summary of the SpongeWorks internal case study descriptions.

Pinios River Basin in Greece

The Pinios River basin is located in Thessaly, central Greece. It is categorized by a Mediterranean climate and is challenged by dry summers. In recent years, Thessaly has experienced extreme flood events, including Storm Medicane Ianos in 2020 and Daniel in 2023. As agriculture covers 50% of the area, this sector is particularly vulnerable to drought and floods.

The Pinios River, Greece's third-longest river, is central to regional water management. The basin has been used as a pilot area for implementation of the EU Water Framework Directive, while approximately 34% of its area is covered by the Natura 2000 network. Main challenges in the basin are surface and groundwater quality and quantity, soil health, flood and soil erosion.

The case study includes four sites: the Agia plain, the Melivia area, the Tavropos area, and the Trikala area. Agia, Melivia and Tavropos represent agricultural contexts and apply sponge measures such as systemic mulching, soil water management, restoring terraces, buffer strips and hedges. Trikala is in an urban setting, implementing narrow riparian forests and a SpongePark for stakeholder engagement and dissemination.

In the SpongeWorks internal workshops that informed this study, the Pinios case study was represented by the expertise of seven local practitioners and three landscape-level stakeholders, sharing insights from years of experience on water-retention measures.

More information can be found on the SpongeWorks website:
https://www.spongeworks.eu/demonstrators/pinios-gr/

Lèze River Basin in France

The Lèze River basin is located in southern France. The river springs in the Pyrenees and flows into the region of Ariège. Characterized by its hilly watershed and the mountain-influenced climate, the region suffers from flash floods, soil erosion and, additionally, water quality issues. Agriculture covers 83% of the basin area, making this sector extremely affected by extreme weather events.

In recent decades, the Lèze basin has been intensively working on addressing the effects on agricultural land. Within SpongeWorks, the region aims for limiting socio-economic impacts of climate effects, improve the ecological status of the water bodies, support water-sensitive agricultural practices and ensure long-term water availability.

The case study includes four sites: Beaumont sur Lèze, Saint Sulpice sur Lèze, Lézat and Le Fossat. All sites represent agricultural contexts, implementing hedges, wood-filtering dams, infiltration ponds and a combination of agro-ecological measures (e.g., pastures, buffer strips, intercropping, mulching, green cover).

In the SpongeWorks internal workshops that informed this study, the Lèze case study was represented by the expertise of two local practitioners and five landscape-level stakeholders, sharing insights on working at the larger scale and with local practitioners.

More information can be found on the SpongeWorks website:
https://www.spongeworks.eu/demonstrators/leze/

Vecht River Basin in the Netherlands and Germany

The Vecht River basin is a cross-border case study spanning 55% in the Netherlands and 45% in Germany. Lying in a lowland European area, the region experiences hot summers and cold winters, leading to large differences in the river's discharge. The river and its tributaries are important for the nearby urban areas, such as Hardenberg (NL) and Bad Bentheim (DE), as well as the agricultural areas, covering 55% of land use. As a cross-border case, the basin is particularly interesting in terms of collaboration between multiple water authorities.

Both urban and rural areas suffer from the impacts of climate change and its weather extremes. Main challenges in the area are surface and groundwater quality and quantity, and mitigating the impact of floods and droughts. Within SpongeWorks, the basin aims to increase water availability during dry periods, increase the basin's resilience across all systemic domains, and increase transboundary collaboration and governance to find long-term basin-wide solutions.

The case study includes two sites in the Netherlands, seven sites in Germany, and one cross-border site. The rural sites are located in Oud-Bergentheim (NL), Rheezerwaterleiding (NL/GER), Itterbeck (GER), Gildehauser Venn (GER), Paradies Kleinringe (GER), Brechte/Feldbecke (GER), Scheerhorn (GER), Laar (GER), Engden (GER). Planned measures contain floodplain restoration, agricultural weirs, trench closures and rock ramps. Hardenberg (NL) represents an urban site, implementing a green cycling lane, including swales, planting trees and removal of pavement.

In the SpongeWorks internal workshops that informed this study, the Vecht case study was represented by the expertise of three local practitioners and two landscape-level stakeholders, sharing experiences from sponge landscape strategies and their governance, as well as local implementation.

More information can be found on the SpongeWorks website:
https://www.spongeworks.eu/demonstrators/vecht/

Table SI1 *Literature scoping for procedural steps in developing catchment sponge strategies. The analysis of the literature steps provided a generic procedure that we used as a basis for the learning framework*

Frameworks					
**Nesshöver et al. 2017**	**Kumar et al. 2020**	**Albert et al. 2021**	**Beek et al. 2022**	**Wickenberg 2024**	**PHASES**
		Co-define setting	Inception		**Define setting**
Dealing with uncertainty, complexity, ambiguity and conflicts to achieve equitable trade-offs;<br>Developing common understanding of multifunctional solutions	Identification of the main hazards	Understand challenges	Situational analysis	Joint understanding & building relations	**Understand challenges**
Ensuring the involvement of multiple stakeholders and a wider public					**Stakeholder engagement**
	(Impact modelling &) Scenario modelling	Create visions and scenarios		Visioning & Roadmap	**Scenarios**
Ensure equitable distribution of benefits and risks	Impact modelling (& Scenario modelling)	Assess potential impacts			**Assessment**
Develop and implement appropriate institutional arrangements/designs	Policy framework for NBS implementation	Develop solution strategies	Strategy building		**Strategies**
Ensure the use of sound multi-disciplinary and trans-disciplinary knowledge, concepts and methods	Co-development			Targeted Collaboration	**Stakeholder integration**
	Selection of the most suitable sets of NBS and socio-institutional measures		Action planning		**Action planning**
Set up long-term investment and financing to reap benefits of NbS					**Financing and business models**
	Test and demonstrate;<br>NbS implementation	Realise (& Monitor)	Implementation		**Implementation**
Ensure environmental targets to be included and monitored	Monitoring (and evaluation) of NbS project	(Realise &) Monitor			**Monitoring**
	(Monitoring and) evaluation of NbS project				**Evaluation**
Evaluation and monitoring for mutual learning across scales				Horizontal Learning	**Reflexive learning**

Table SI2 *Literature scoping for procedural steps in developing local* <u>*sponge measures*</u>*. The analysis of the literature steps provided a generic procedure that we used as a basis for the learning framework*

Frameworks					
**Raymond et al. 2017**	**Beek et al. 2022**	**de Lima et al. 2022**	**Wren et al. 2022**	**Collier et al. 2023**	**PHASES**
	Inception		Initiation		**Define setting**
Identify problem or an opportunity	Situational analysis	Research and development	Understand catchment		**Understand challenges**
				Co-Production	**Stakeholder engagement**
		Planning		Planning	**Project planning**
Select and assess NbS and related actions				Impact assessment	**Assessment**
	Strategy building	Conceptual design	(Monitor &) manage	Governance	**Strategies**
Design NbS implementation processes	Action planning	Detailed design	Select measures; design	Technical solutions	**Action planning**
				Financing and business models;	**Financing and business models**
				Nature-based enterprises	
Implement NbS	Implementation	Construction	Construct & implement	Delivery	**Implementation**
Monitor (and evaluate) co-benefits across all stages		Monitoring	Monitor (& manage)	Stewardship	**Monitoring**
(Monitor and) evaluate co-benefits across all stages		Evaluation			**Evaluation**
		Adjustment			**Adjustment**
Transfer and upscale NBS					**Share learnings**
				Reflexive monitoring	**Reflexive learning**

Table SI3 *Workshop 1:* <u>*Sponge Landscape Strategy*</u> *results of key activities in each phase. Right column: Potential Framework Adaptation according to the results*

**Phases**	**Building Blocks**	**Workshop results**
Ignite Planning Process	Define aim & scope	Define natural borders in which you are working
		Define why we want this
		Define the time scale
	Identify key stake- & knowledge holders	Identify who/what is impacted and in what way(s) --> comment from Kelsey: I think this could come as the first step before define aim/scope
		Select from pool of stakeholders influential and knowledgeable actors
		Identifying change stakeholders
		Identify barriers to participation

	Agree on process & mandate	Flexible processes with individual preparatory phases, not long but effective and focused engagement
		Include processes that are flexible/adaptable to stakeholder capacity/interest to engage
		Communicating the process
Co-generate system understanding	Develop shared understanding	Identify knowledge sources, including local or traditionally overlooked sources of knowledge
		Analyse existing knowledge, historical events and key projections
		Knowing what already existing in the territory
	Identify key challenges	Identify policy and Infrastructure gaps
		Demonstrate/identify how challenges relate or are connected
		Analyse and agree on key challenges based on the understanding
		Do not point the finger at those responsible for the current situation
	Explore direct & indirect drivers	Identify cross-sectoral interdependencies (CLDs at some stage??? to identify the drivers))
		Identify contextual factors that influence challenges
Co-perform spatial planning	Create Scenarios	Baseline/do nothing vs...
		Be based on historical events (no uncertainty)
		Define what an extreme event is based on indices that are comparable under different bioclimatic regions
	Explore scenario impacts	Identify who/what is likely to be impacted in the future (2050 etc.) and how
		Scenario have economic impact?
		Identify key sectors to be impacted and key groups to be impacted
		Integrated approach - must involve the various stakeholders
	Develop action plans	Connect back to aim and scope
		Set the targets of the plans, Prevent impact X on group Y
Co-develop implementation strategy	Understand barriers & bridges	Identify power relations
		Clarify different administrative levels
		Identify key layers and regulatory structures
		Understand coalitions (formal-informal)
		Work on sector and not just in localisation
	Explore governance options	Learn from previous failures to design new governance mechanisms
		Understand how the structure that existis can fascilitate structuring the strategy
		Ensure maximum alignment possible with existing strategies
		Administrative simplification
	Develop roadmaps	Roadmap including all stakeholders
		Suggest different road maps
		Get representative, influential, knowledgeable stakeholders of all levels of hierarchy on Board. Ensure interrelationships amongst them are fair
Co-implement strategy & learning	Execute implementation strategy	Establish responsibilities
		Identify process indicators for monitoring
		Involve all key actors, make it representative, ensure commitment
		Ensure regulatory transformations are in place to accelerate
	Establish monitoring of changes	Collect data before changes
		Establish a well-documented baseline
		Define simple, plausible, yet representative and meaningful indicators
	Employ adaptive governances	Ensure close collaboration with decision makers and assessors of policies' implementation impact

Share insights & experiences	Establish new policies & advocacy	Determine the most effective/appropriate level for policy implementation
		Create advocacy messaging with impacted stakeholders
		Adapt-reorganise to offer an integrated approach of all potentially involved policy actors
	Disseminate adaptation potential	Narrative storytelling
		Specify that each region must take account of its local constraints
		Use appropriate semantics
		Fact sheets, successful stories
	Establish communities of practices	Identify existing communities
		Built on what's in place and identify task force groups for individual issues

Table SI4 *Workshop 1:* <u>*Local Sponge Measures*</u> *results of key activities in each phase. Right column: Potential Framework Adaptation according to the results*

Phases	Building Blocks	Workshop results
Ignite Planning Process	Define aim & scope	Clearly articulate the objectives
		Establish a general understanding of the project timeline and key actions
		Scope determination of own project in relation to SpongeWorks goals and cooperation
		Relate the project objectives to local and regional realities
		Align with municipal urban planning policies and sustainability goals
		Share a vision of what success looks like (benefits) and how to achieve financial sustainability
		Clearly define costs & benefits to particular implementers
		Define an intervention framework
		Address urban water retention challenges
		Defining the ambition of climate adaptation in the project, based on our internal guidelines (Green & Healthy yardstick)
		In General: theory versus reality. in terms of process, we can prepare everything well, but reality is unruly and often just different
	Identify key stake- & knowledge holders	Join project meetings, in person if possible, and build relations with close partners
		Understanding expectations and existing collaborations
		Exploit existing partner networks
		Try to find common ground among partners
		Linking SpongeWorks with other 'similar' initiatives
		Recognise potential opponents and critics
		Understanding the project area and the stakeholders located in the area. Assess their interests (and conflicts)
		Define which stakeholders? A large open call for projects open to individuals, cities, farmers? Or do we designate which stakeholders might be interested?
	Agree on process & mandate	Follow an inclusive process and encourage participation
		Needs support from local authorities
		Attention needed for alignment with governance (including changes of directors)
		When having a common understanding of the project timeline, after and having a general understanding of the partner motivations, you discuss how to work together
		Determine who has what role in project and what the expectations are
		Deciding together on how to best take the next steps
		Get support from stakeholders for a project plan, in order to get commitment from the municipal council

		Try not "just" implement the project, but where possible connect it to ongoing existing transformations
		Need to be clear about property rights.
		If public funds are distributed, need a transparent selection process
Co-perform implementation planning	Design implementation & maintenance	Define who has the responsibility to organise a sponge measure plan
		Needs local consultation
		Define how to implement a sponge strategy: convince one by one urban stakeholders, farmers, owners? or make consultation phase?
		Define who will make the maintenance. For wood dams, for example, who will verify that all is ok? and in 20 years? Is there a risk to break down?
		Start with localised measures - adopters need to be convinced about their own costs & benefits, rather than the 'big picture'.
		Evaluate the resilience of such measure on flooding
		Set up a clear framework of collaboration: what are the commitments, responsibilities & benefits?
		Sort out all project requirements to be implemented by measures
		Plan integration with existing infrastructure
		Do note "take this over" as a project, but let the ownership be with the experts on the ground
		Plan integration with existing practices (e.g., agroecological practices)
	Assess potential impacts	Evaluate social and economic benefits
		Evaluate the risk if all goes wrong: wood dams that break, cover crops that don't succeed or impact the production negatively
		Clearly communicate potential risks, expected costs & benefits, and related timelines
		Evaluate the risks now and in 20, 50 years
		Performing a risk analysis and act upon it
		Quantitative data on flood resilience
		Let your implementers and stakeholders define impact and allow them to co-assess
	Obtain approval & perform procurement	Permits and plan approval take time (especially in case of objections); ensure support
		Establish agreements with stakeholders for long-term project sustainability
		Framework of procurement needs to be laid out clearly in advance
		Secure necessary permits from municipal and regional authorities
		Get approval of the plan from the right stakeholders, including internal management and council
Co-implement measures & maintenance	Perform construction	Final design and technical documents, construction party
		Ensure coordination with municipal construction teams
		Assist private implementers (e.g., farmers) to ensure correct implementation.
		Let this be done by partners that are already doing this
		How to proceed when there is a forest owner on one side who supports a wooden dam to raise their waterline, and a cultivated plot on the opposite side that doesn't want to lose its income?
	Implement maintenance	Establish a municipal maintenance schedule for sponge measures
		Maintenance must be thought during the design phase
		Final design and required maintenance should be fine-tuned together with those who maintain it
		Assist private implementers (e.g., farmers) for successful maintenance
		Collect feedback on problems encountered and address them
Monitor & Learn	Ex-ante assessment	Importance of ex-ante estimation (hypothesis) based on existing knowledge
		Map existing green infrastructure and soil conditions
		Should be a part of the organisation's culture and policies

	Accompanying assessment	Organise regular progress meetings with stakeholders to assess effectiveness
		Should be a part of the organisation's culture and policies
	Post-ante assessment	Collect stakeholder feedback and adjust
		Compare pre- and post-implementation data to measure improvements in water retention
		Should be a part of the organisation's culture and policies
Share insights & experiences	Establish new policies & advocacies	Invite local decision makers to see the implementation and the results
		Provide evidence to policymakers
	Disseminate adaptation potential	Multimedia content
		Awareness campaigns
		Videos
		Figures
		Videos for student also, which can be used in lessons, like one hour documentary
		Proven and data-driven effectiveness
		Visits
		Identify local 'champions' who are already convinced and use them as 'ambassadors'
		Communicate proven benefits & success stories to local society
		Use storytelling to share
	Establish communities of practices	Organise site visits
		Peer-to-peer learning
		Share (initial) results as early as possible; may help others
		Like a WhatsApp group where people can ask questions or share some success stories
		Think about what can be interesting and not lead to stakeholder fatigue
		Link with existing CoPs and networks -don't try to invent new ones
		Ensure multilingual accessibility

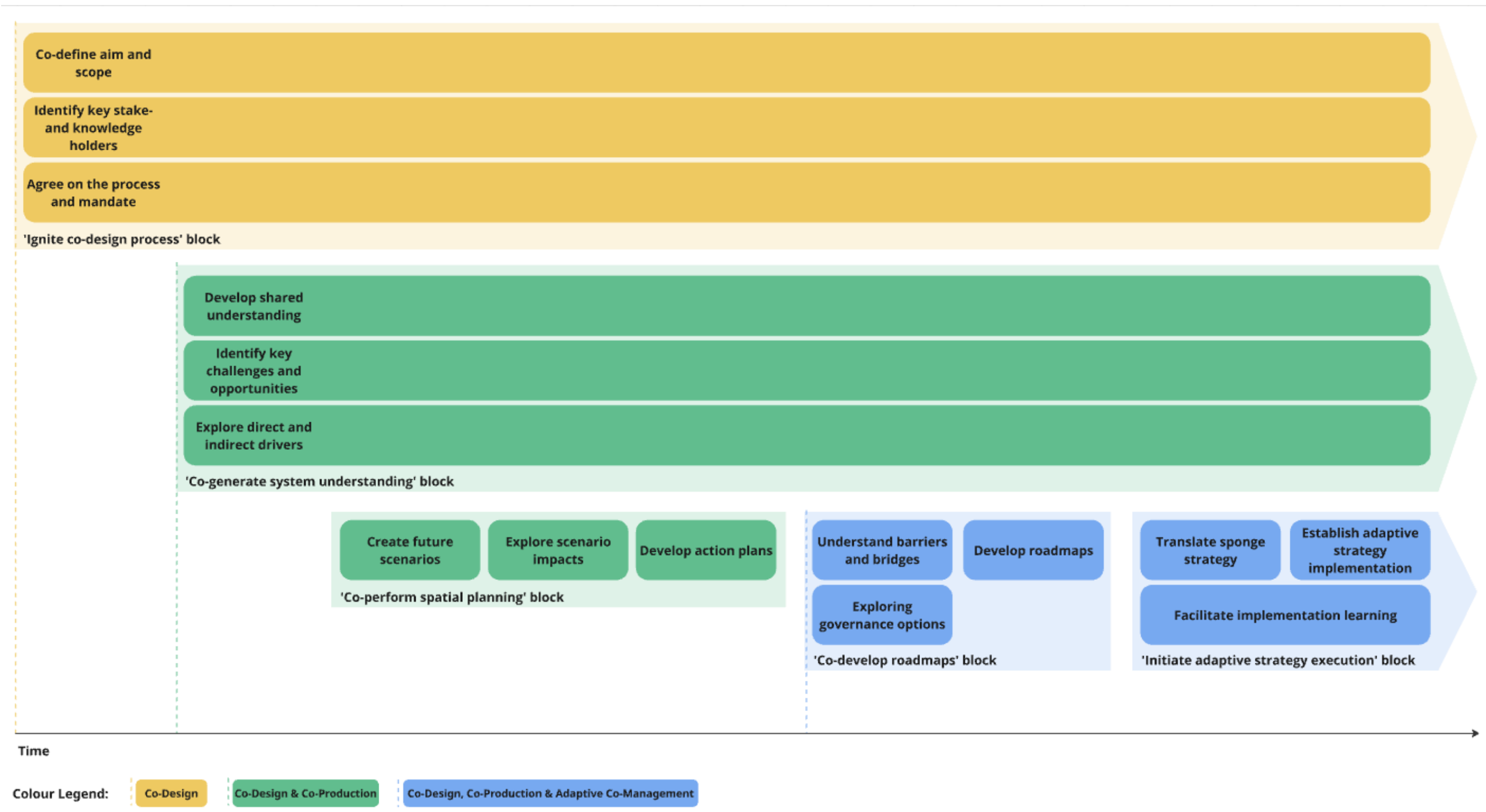

Fig. SI1 *Example of a sequential development process for sponge landscape strategies, going from the top left to the bottom right*

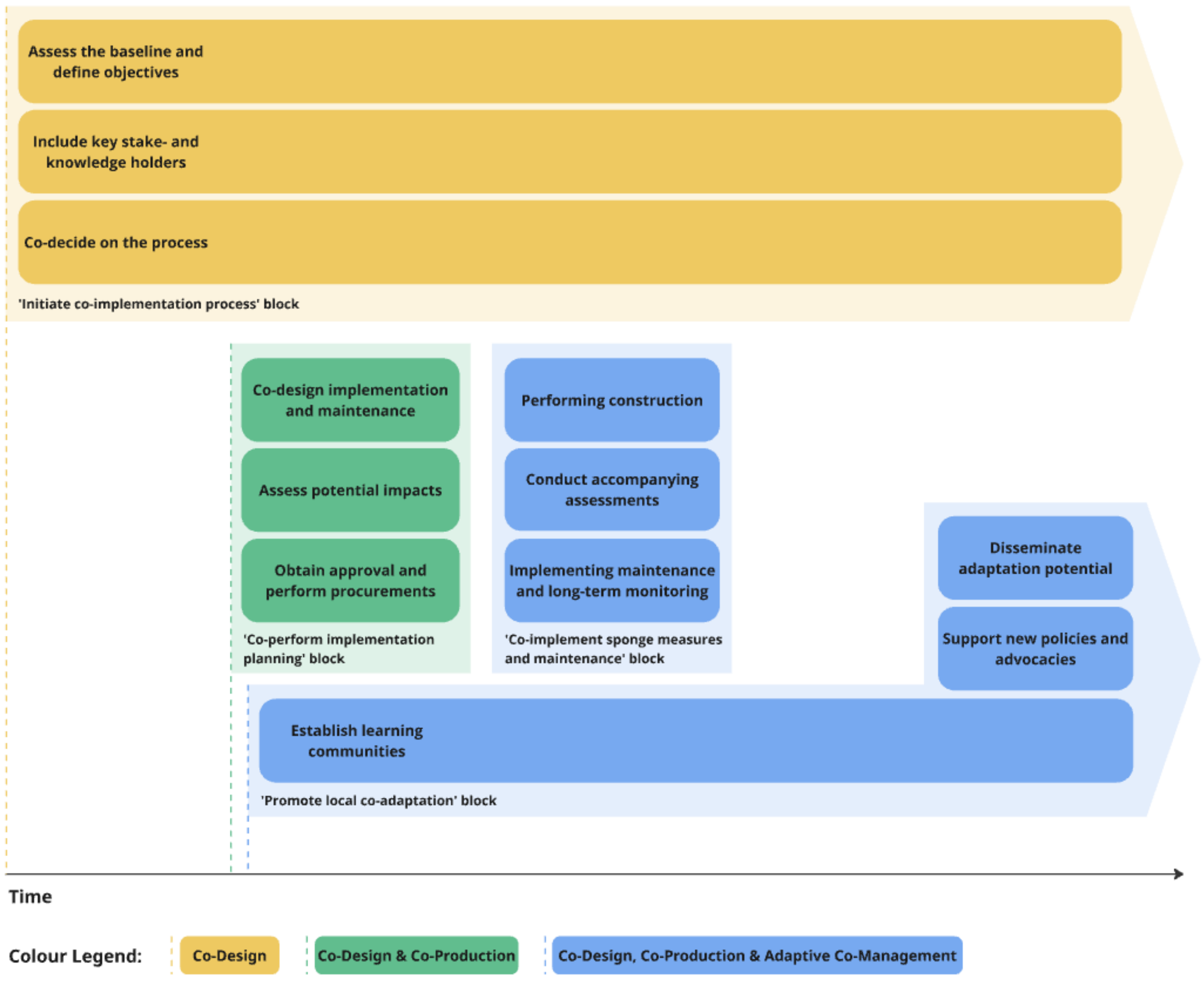

Fig. SI2 *Example of a sequential development process for local sponge measures, going from the top left to the bottom right*

References

Albert C, Brillinger M, Guerrero P, Gottwald S, Henze J, Schmidt S, Ott E, Schröter B (2021) Planning nature-based solutions: Principles, steps, and insights. Ambio 50:1446–1461. https://doi.org/10.1007/s13280-020-01365-1

Beek, E.v, Nolte, A.J., Maat, J.t, Fanesca - Sanchez, M., Asselman, N. Gehrels, H. (2022) Strategic Water System Planning: A framework for Achieving Sustainable, Resilient and Adaptive Management, Delft, Netherlands. https://cms.deltares.nl/assets/common/downloads/Deltares-Strategic-Water-Systems-planning_v1.1-December-2022-LOW_RES_compressed.pdf

Collier MJ, Frantzeskaki N, Connop S, Dick G, Dumitru A, Dziubała A, Fletcher I, Georgiou P, Hölscher K, Kooijman E, Lodder M, Madajczyk N, McQuaid S, Nash C, Osipiuk A, Quartier M, Reil A, Rhodes M-L, Rizzi D, Vandergert P, van Sijpe K de, Vos P, Xidous D (2023) An integrated process for planning, delivery, and stewardship of urban nature-based solutions: The Connecting Nature Framework. Nature-Based Solutions 3:1-10. https://doi.org/10.1016/j.nbsj.2023.100060

de Lima APM de, Rodrigues AF, Latawiec AE, Dib V, Gomes FD, Maioli V, Pena I, Tubenchlak F, Rebelo AJ, Esler KJ, Oen AMP, Ramírez-Agudelo NA, Bosch ER, Singh N, Suleiman L, Hale SE (2022) Framework for Planning and Evaluation of Nature-Based Solutions for Water in Peri-Urban Areas. Sustainability 14:1-15. https://doi.org/10.3390/su14137952

Kumar P, Debele SE, Sahani J, Aragão L, Barisani F, Basu B, Bucchignani E, Charizopoulos N, Di Sabatino S, Domeneghetti A, Edo AS, Finér L, Gallotti G, Juch S, Leo LS, Loupis M, Mickovski SB, Panga D, Pavlova I, Pilla F, Prats AL, Renaud FG, Rutzinger M, Basu AS, Shah MAR, Soini K, Stefanopoulou M, Toth E, Ukonmaanaho L, Vranic S, Zieher T (2020) Towards an operationalisation of nature-based solutions for natural hazards. Sci Total Environ 731:1-23. https://doi.org/10.1016/j.scitotenv.2020.138855

Nesshöver C, Assmuth T, Irvine KN, Rusch GM, Waylen KA, Delbaere B, Haase D, Jones-Walters L, Keune H, Kovacs E, Krauze K, Külvik M, Rey F, van Dijk J, Vistad OI, Wilkinson ME, Wittmer H (2017) The science, policy and practice of nature-based solutions: An interdisciplinary perspective. Sci Total Environ 579:1215–1227. https://doi.org/10.1016/j.scitotenv.2016.11.106

Raymond CM, Frantzeskaki N, Kabisch N, Berry P, Breil M, Nita MR, Geneletti D, Calfapietra C (2017) A framework for assessing and implementing the co-benefits of nature-based solutions in urban areas. Environmental Science & Policy 77:15–24. https://doi.org/10.1016/j.envsci.2017.07.008

Wickenberg B (2024) Collaborating for nature-based solutions: bringing research and practice together. Local Environment 29:118–134. https://doi.org/10.1080/13549839.2023.2254797

Wren E, Barnes M, Kitchen A (2022) The natural flood management manual. CIRIA, London
